# Photocatalytic Efficiency of g-C_3_N_4_/Graphene Nanocomposites in the Photo-Assisted Charging of the Li-Ion Oxygen Battery

**DOI:** 10.1021/acsomega.3c07546

**Published:** 2023-11-23

**Authors:** Nilay Kaçar, Meltem Çayirli, Reşat Can Özden, Ersu Lökçü, Mustafa Anik

**Affiliations:** Department of Metallurgical and Materials Engineering, Eskisehir Osmangazi University, Eskisehir 26040, Turkey

## Abstract

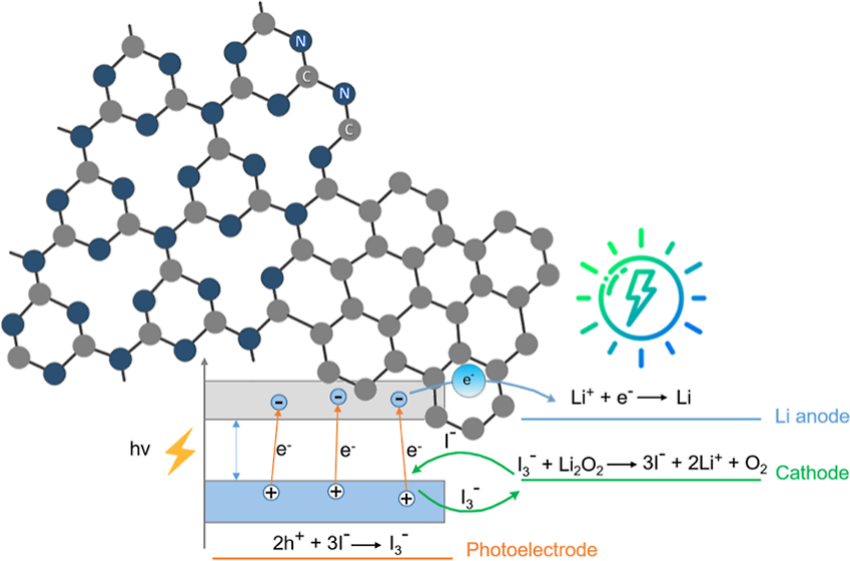

In this work, we aimed to synthesize an effective nanocomposite photocatalyst for the photo-assisted charging of the Li-ion oxygen battery. Initially the graphene films were synthesized by chemical vapor deposition, and subsequently, g-C_3_N_4_/graphene nanocomposites were synthesized by thermal reduction as photocatalysts. FTIR spectra analysis showed that novel C=C bonds can form between g-C_3_N_4_ and graphene films during the synthesis process. The photocurrent measurements indicated that the presence of graphene considerably contributed to the visible light utilization and photocatalytic efficiency of g-C_3_N_4_. This contribution was also revealed by the UV–vis diffuse reflectance spectra measurements, which showed that the incremental addition of the graphene reduced the optical band gap of the nanocomposite incrementally. The photocatalyst performance of the g-C_3_N_4_/graphene nanocomposite was also observed in the photo-assisted charging tests of the Li-ion oxygen battery, and the presence of 2D graphene in the structure improved the effectiveness of g-C_3_N_4_ in the reduction of the charging potential, especially at high current densities.

## 1 Introduction

The photo-assisted charging of the Li-ion oxygen batteries with the aid of triiodide/iodide (I_3_^–^/I^–^) redox shuttling by using a photocatalyst was proposed to reduce the extended charging potential arising from the sluggish oxidation kinetics of the low-conducting Li_2_O_2_.^1^ The generated photovoltage enables the reduction of the charging potential down to a conduction band (CB) edge potential of the photocatalyst at low charging current densities.^1^ A critical requirement for the photocatalyst is that the valence band (VB) edge potential must be higher than the redox potential of the I_3_^–^/I^–^ couple to drive the oxidation of I^–^ ions to I_3_^–^ ions. Dye-sensitized TiO_2_,^1^ g-C_3_N_4_,^2^ TiN/TiO_2_/carbon cloth,^3^ Au/TiO_2_ nanotubes,^4^ TiO_2_/Fe_2_O_3_,^5^ WO_3_/g-C_3_N_4_,^6^ and rGO/g-C_3_N_4_^7^ were synthesized as photocatalysts for the photo-assisted charging of the Li-ion oxygen batteries. In addition to the thermodynamic requirements, the visible light harvesting efficiency of the photocatalyst is an another criterion in the reduction of the charge potential at high charging current densities.^7^

Two-dimensional (2D) materials and their composites are expected to make a significant impact on the advancement of the energy conversion and storage technologies.^8,9^ Graphitic carbon nitride (g-C_3_N_4_), a 2D nonmetallic semiconductor, is a very promising photocatalyst owing to its convenient light harvesting band gap (2.7 eV), good physicochemical stability, and easy and low-cost synthesis.^10−14^ However, the severe recombination of the photogenerated electron–hole pairs, low surface area, and low electrical conductivity restrict its application.^15^ The synthesis of g-C_3_N_4_-based nanocomposites with the carbonaceous nanomaterials is accepted as the most effective pathway to overcome these handicaps since they are the most compatible constituents of the nanocomposites due to their similar carbon network and sp^2^-conjugated π structure.^16−24^

Graphene, a 2D single-layer sheet of sp^2^-hybridized carbon atoms, attracts worldwide research interest due to its exceptional physical properties.^25^ Graphene finds large practical applications in microelectronics, optoelectronics, and energy storage.^26^ Chemical vapor deposition (CVD) is recognized as the most suitable synthesis route to get high quality (with low structural defects) graphene.^27^

In this work, following the efforts on the development of the effective photocatalyst for the Li-ion oxygen battery, a novel nanocomposite was synthesized. Initially, the few layer graphene films were synthesized on the copper substrate by CVD. Then, the graphene films were mixed with melamine (C_3_H_6_N_6_) and g-C_3_N_4_/graphene nanocomposites were obtained by thermal reduction at different graphene/g-C_3_N_4_ ratios. After structural and optical characterizations of the nanocomposites, the best nanocomposite composition was determined and it was used as a photocatalyst in the photo-assisted charging of the Li-ion oxygen battery. The improved photocatalytic activity was obtained in the g-C_3_N_4_/graphene nanocomposites since the high quality graphene brought about an increase in both the surface area and electrical conductivity of g-C_3_N_4_. Additionally, the presence of graphene caused a redshift and narrowing of the band gap slightly in the semiconductor nanocomposites.

## 2 Experimental Section

### 2.1 Synthesis Methods

The details of the preparation of the palladium-loaded porous reduced graphene oxide (Pd@rGO nanostructure) cathodes (oxygen electrodes) were reported in our previous work.^7^

Graphene was synthesized by CVD according to the procedure provided in Figure S1 on the Cu foil substrate (Sigma-Aldrich, 25 μm thickness, 99.999% purity). After the synthesis process, the Cu substrate was dissolved in 50% HCl solution including 0.5 M FeCl_3_ and the graphene films were collected in high purity ethanol. Since the graphene films were covering the Cu foil substrate surface continuously without any damage, the surface area of the graphene film was assumed to be equal to the surface area of the Cu foil substrate. For the characterizations of the graphene films, the Cu foil was coated with the poly methyl methacrylate (PMMA) on the spin coater and then the copper substrate was dissolved. PMMA was also cleaned with acetone/isopropanol combinations after the coated graphene film was transferred to either SiO_2_/Si or transmission electron microscopy (TEM) grit.

In order to prepare nanocomposites, melamine and graphene were mixed in ethanol at 50 °C until all the ethanol evaporates. After complete drying, melamine and graphene were put into a crucible (30 mL) under continuous Ar flow (1.2 L min^–1^) and heated up to 550 °C at a rate of 3 °C min^–1^ and then kept at this temperature for 3 h. Subsequently, g-C_3_N_4_/graphene nanocomposites were collected after cooling to R.T. Nanocomposite compositions were determined according to the ratio of graphene surface area to g-C_3_N_4_ weight (cm^2^ graphene/mg g-C_3_N_4_).

### 2.2 Electrode Preparations and Electrochemical Measurements

The working electrode was prepared by loading the nanocomposite on ITO glass (10 Ω cm^–2^) by a spin coater and then drying at 100 °C for 12 h in a vacuum-desiccator. The photoelectrochemical measurements were made in a conventional three-electrode cell with a platinum wire as the auxiliary electrode and an Ag/AgCl (saturated KCl) as the reference electrode on a Gamry Reference 3000 workstation in a spectral cell containing 0.1 M KCl buffered by 0.1 M K_2_HPO_4_ to pH 7. The photocurrents were measured by the linear sweep voltammetry technique, and the semiconductor loading was 0.05 mg cm^–2^. A solar simulator (A-type 150 W, 1–3 SUN, Xenon lamb, AMO filters; 400–700 nm wavelength) was used as the light source. The semiconductor loading was 0.8 mg cm^–2^ for the Mott–Schottky measurements.

Photo-assisted charging of the Li-ion oxygen battery was carried out by a homemade cell in an oxygen cabin, which had 1 bar positive oxygen pressure during the measurements, as shown in our previous work.^7^ The cell assemblage was carried out in an Ar-filled glovebox with H_2_O and O_2_ levels less than 0.1 ppm. Lithium metal was used as both counter and reference electrodes, and the glass microfiber filter (Whatman) was used as a separator. The oxygen electrode was prepared by mixing the porous Pd@rGO/Super P carbon black/PVDF (80:10:10 wt %) in NMP, and then, the slurry was coated onto one side of 16 mm diameter GDL (TGP-H-060) with a loading rate of 0.1 mg cm^–2^. The semiconductor nanocomposite was coated on the other side of GDL with a loading rate of 0.05 mg cm^–2^ as a photoelectrode. Before the battery assembly, the electrodes were dried in a vacuum oven at 100 °C overnight. 0.5 M LiClO_4_ and 0.05 M LiI dissolved in TEGDME were used as an electrolyte for photo-assisted charging. LiI was excluded if charging was conducted without illumination. The discharge and charge tests were performed galvanostatically and the discharge cutoff potential was 2.0 V_Li+/Li._ The charge cutoff potentials were 3.6 V_Li+/Li_ and 4.2 V_Li+/Li_ for the photoassisted and dark charging, respectively. The current densities changed between 10 mA g^–1^ (10^–3^ mA cm^–2^) and 500 mA g^–1^ (5 × 10^–2^ mA cm^–2^).

### 2.3 Structural Characterizations

X-ray diffraction (XRD) analyses were performed on a PANalytical Empyrean diffractometer with Cu Kα radiation at a scanning rate of 2° min^–1^. The morphologies were examined with a ZEISS Ultraplus scanning electron microscope. FTIR measurements were conducted by a PerkinElmer Spectrum Two. The graphene film morphology was characterized using a JEOL JEM 2010F transmission electron microscope running at 200 kV and atomic force microscopy (AFM, Veeco Innova). UV/vis spectra were recorded by Cary 5000 UV/vis/NIR spectrometer with a diffuse reflectance accessory between 200 and 800 nm. The Raman analysis was carried out by Raman spectroscopy (RENISHAW RAMAN inVia microscope) with an excitation wavelength of 532 nm.

## 3 Results and Discussion

### 3.1 Structure and Morphology

The normalized Raman spectra from the synthesized graphene film are provided in Figure S2a. Two main peaks can be assigned in the Raman spectrum. The G (∼1582 cm^–1^) and 2D (∼2694 cm^–1^) bands are attributed to the sp^2^ hybridizations of the C atoms.^28^ The 2D band is sensitive to the number of layers of graphene films.^28^ The 2D/G intensity ratio is 3.2 in Figure S2a, in which the synthesized graphene looks to have only a few layers. The absence of the D band in Figure S2a indicates that the graphene films are synthesized without any defect. The scanning electron microscopy (SEM) and TEM images of the synthesized graphene on the TEM grit are presented in Figure S2b,c, respectively. These images reflect the typical few-layer graphene morphology. It is also understood from the images that there are occasional wrinkles and overlaps. Similar characteristics of the graphene film can also be observed in the AFM image in Figure S2d.

**Figure 1:**
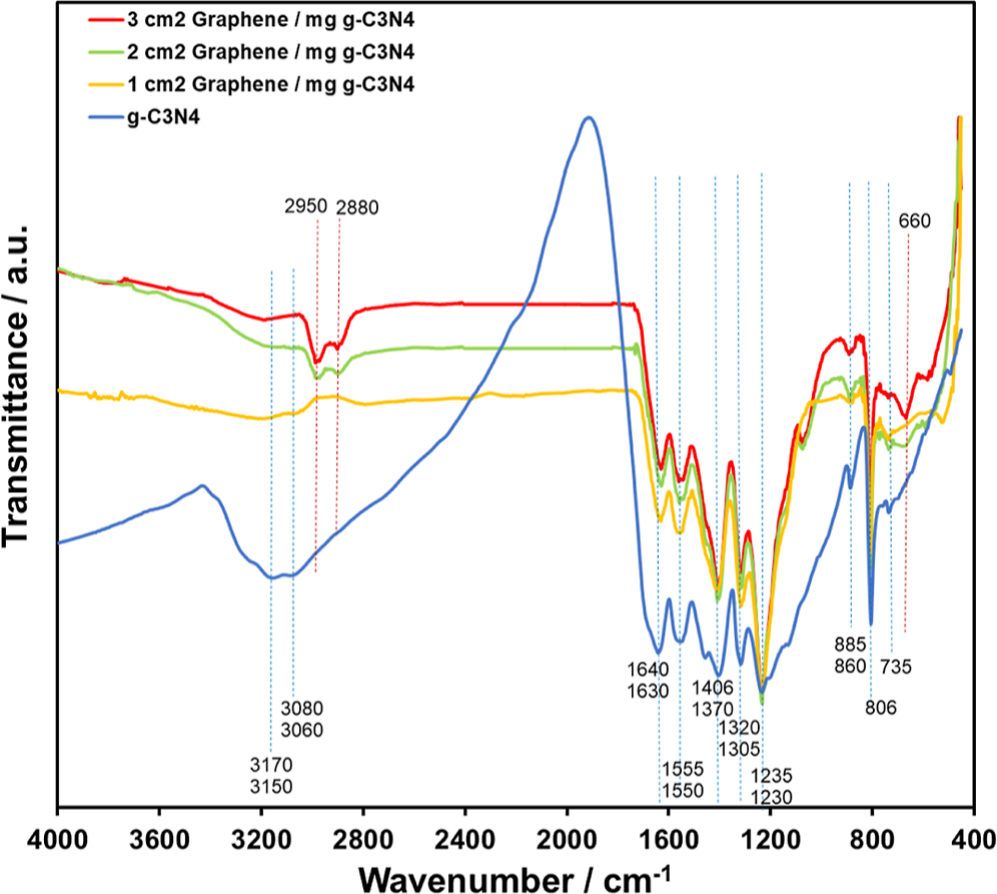
FTIR spectra of pure g-C_3_N_4_, 1 cm^2^ graphene/mg g-C_3_N_4_, 2 cm^2^ graphene/mg g-C_3_N_4_, and 3 cm^2^ graphene/mg g-C_3_N_4_.

The composition of the g-C_3_N_4_/graphene nanocomposites are determined according to the total surface area of graphene (cm^2^) per weight of g-C_3_N_4_ (mg) present in the nanocomposite structure. The nanocomposites with three different compositions are synthesized: 1 cm^2^ graphene/mg g-C_3_N_4_, 2 cm^2^ graphene/mg g-C_3_N_4_, and 3 cm^2^ graphene/mg g-C_3_N_4_. The structural changes in the synthesized nanocomposites are identified by FTIR spectra (normalized), as shown in Figure 1. In order to present clearly, peaks belonging to pure g-C_3_N_4_ and nanocomposites (novel ones) are shown by blue and red dashed lines, respectively, in Figure 1. The broad absorption peaks between 3000 and 3200 cm^–1^ are associated with N=H stretching (due to residual amino groups or absorbed water) in pure g-C_3_N_4_.^29^ Spectrum peaks located between 1200 and 1650 cm^–1^ are due to the stretching modes of CN heterocycles.^29^ Peaks located in the range change from 735 to 806 cm^–1^ and at 885 cm^–1^ are attributed to the triazine ring stretching and N–H band deformation modes, respectively.^29^ The synthesized nanocomposites have all of the characteristic peaks of pure g-C_3_N_4_. Novel peaks (marked by red dashed lines) in nanocomposites located in the range between 2880 and 2950 cm^–1^ (medium strength) and at 660 cm^–1^ (strong) can be attributed to the C–H stretching vibrations probably due to the interaction of graphene with the residual amino groups or absorbed water, and the C=C bond bending vibrations, respectively, in Figure 1. The strong peak appearing belonging to the C=C bond shows the probable bond formation between g-C_3_N_4_ and graphene during the decomposition of melamine to g-C_3_N_4_ at 550 °C as reported previously.^7^

**Figure 2:**
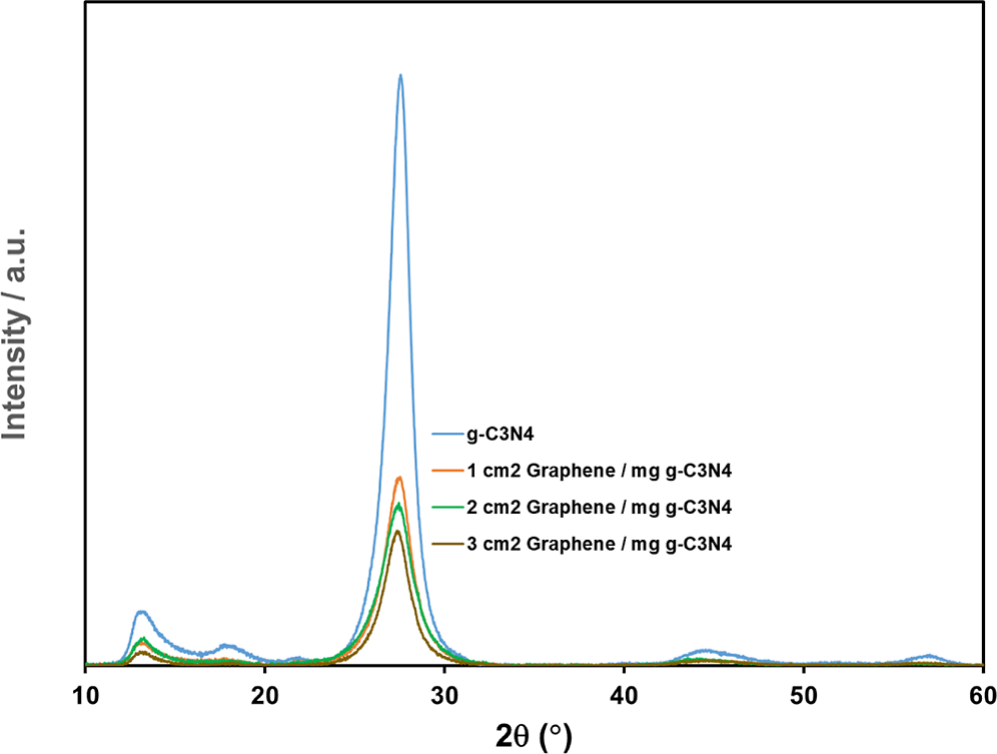
XRD patterns of pure g-C_3_N_4_, 1 cm^2^ graphene/mg g-C_3_N_4_, 2 cm^2^ graphene/mg g-C_3_N_4_, and 3 cm^2^ graphene/mg g-C_3_N_4_.

Figure 2 shows the XRD patterns of the pure g-C_3_N_4_ and g-C_3_N_4_/graphene nanocomposites. A strong characteristic (002) peak at 27.6° in the pure g-C_3_N_4_ pattern is associated with the layered structure.^17^ The peak at around 13.2° (100) in the pure g-C_3_N_4_ pattern in Figure 2 corresponds to the in-plane ordering of tri-*s*-triazine units.^28^ All the nanocomposites have almost the same characteristic peaks as those of pure g-C_3_N_4_ in Figure 2.

The morphologies of the synthesized 1 cm^2^ graphene/mg g-C_3_N_4_, 2 cm^2^ graphene/mg g-C_3_N_4_, and 3 cm^2^ graphene/mg g-C_3_N_4_ nanocomposites are provided in Figure S3. Morphologies of the nanocomposites are very similar to that of pure g-C_3_N_4_, which has a characteristic slate-like stacked lamellar microstructure.^7^ No clear change is observed in the nanocomposite morphologies since the amount of CVD graphene is considerably small in the nanocomposite structure.

### 3.2 Optical Properties

**Figure 3:**
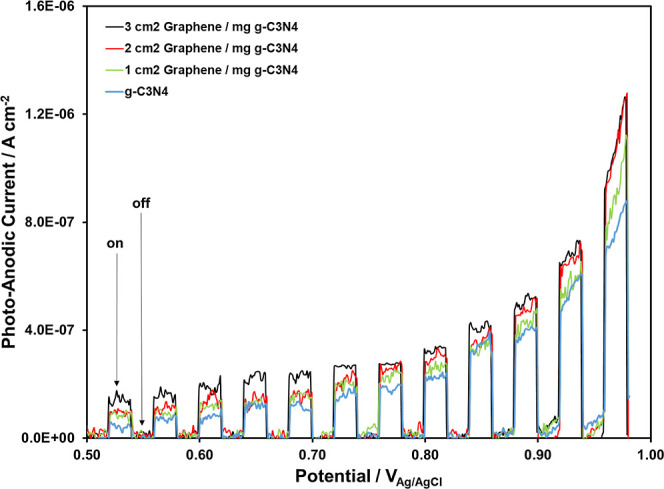
Photoanodic currents of pure g-C_3_N_4_, 1 cm^2^ graphene/mg g-C_3_N_4_, 2 cm^2^ graphene/mg g-C_3_N_4_, and 3 cm^2^ graphene/mg g-C_3_N_4_.

The photoanodic currents, via on–off cycles under visible-light irradiation, are obtained for all the synthesized nanocomposites and pure g-C_3_N_4_ by the linear sweep voltammetry techniques, as shown in Figure 3. The incremental incorporation of graphene results in an increase in the photocurrents. Obviously, the presence of graphene provides more efficient visible light utilization and enhances the photocatalytic efficiency of g-C_3_N_4_.

**Figure 4:**
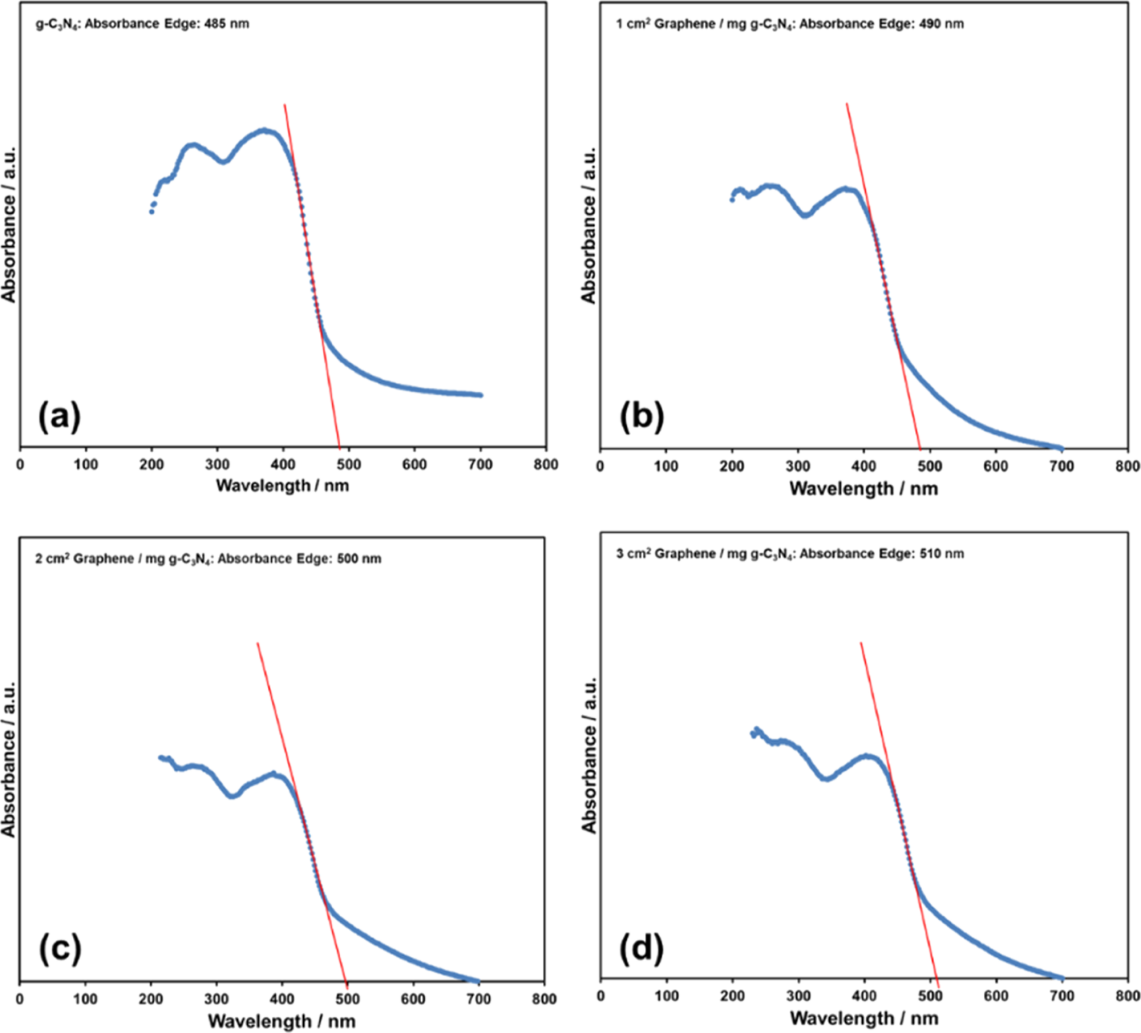
Absorption edges of (a) pure g-C_3_N_4_, (b) 1 cm^2^ graphene/mg g-C_3_N_4_, (c) 2 cm^2^ graphene/mg g-C_3_N_4_, and (d) 3 cm^2^ graphene/mg g-C_3_N_4_.

**Figure 5:**
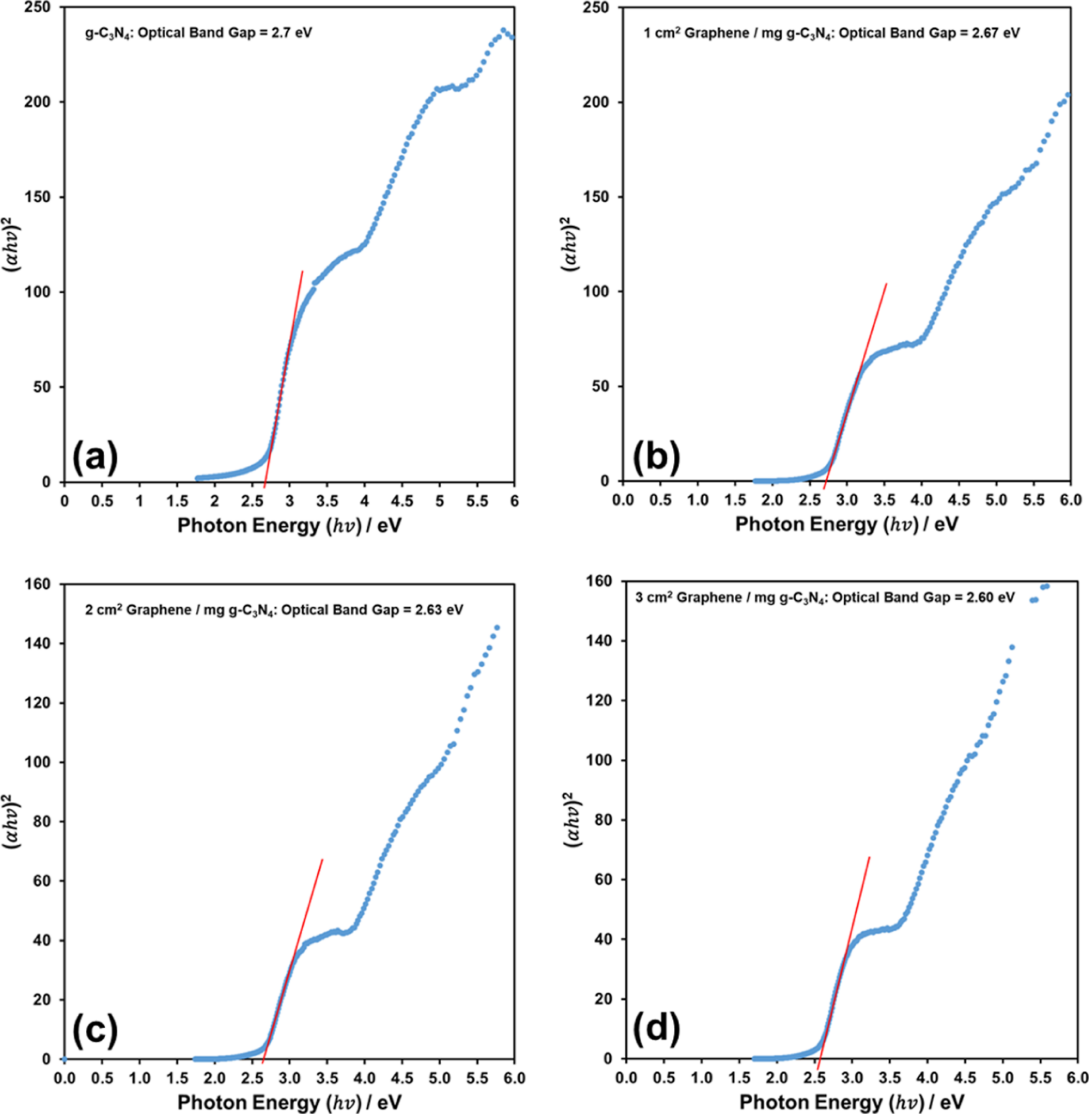
Optical band gaps of (a) pure g-C_3_N_4_, (b) 1 cm^2^ graphene/mg g-C_3_N_4_, (c) 2 cm^2^ graphene/mg g-C_3_N_4_, and (d) 3 cm^2^ graphene/mg g-C_3_N_4_.

The UV–vis diffuse reflectance spectra of pure g-C_3_N_4_ and nanocomposites are given in Figure 4. The absorption band edge of g-C_3_N_4_ is 485 nm and it increases slightly to 490, 500, and 510 nm for 1 cm^2^ graphene/mg g-C_3_N_4_, 2 cm^2^ graphene/mg g-C_3_N_4_, and 3 cm^2^ graphene/mg g-C_3_N_4_, respectively. The optical band gaps (*E*_g_) can be obtained by the Tauc plot,^30^ as in Figure 5, and they are 2.7, 2.67, 2.63, and 2.60 eV for g-C_3_N_4_, 1 cm^2^ graphene/mg g-C_3_N_4_, 2 cm^2^ graphene/mg g-C_3_N_4_, and 3 cm^2^ graphene/mg g-C_3_N_4_, respectively. It was reported previously that the incorporation of the nanocarbons to g-C_3_N_4_ causes a redshift in the absorption band edges of g-C_3_N_4_ and thus the absorption band edges shift to the higher wavelengths and then the corresponding optical band gaps are narrow.^7^ The redshift in the nanocomposites is attributed to the formation of a novel chemical bonding during the synthesis of the nanocomposites.^7^Figures 4 and 5 show that a similar redshift and band gap narrowing are also observable slightly in the graphene/g-C_3_N_4_ nanocomposites.

**Figure 6:**
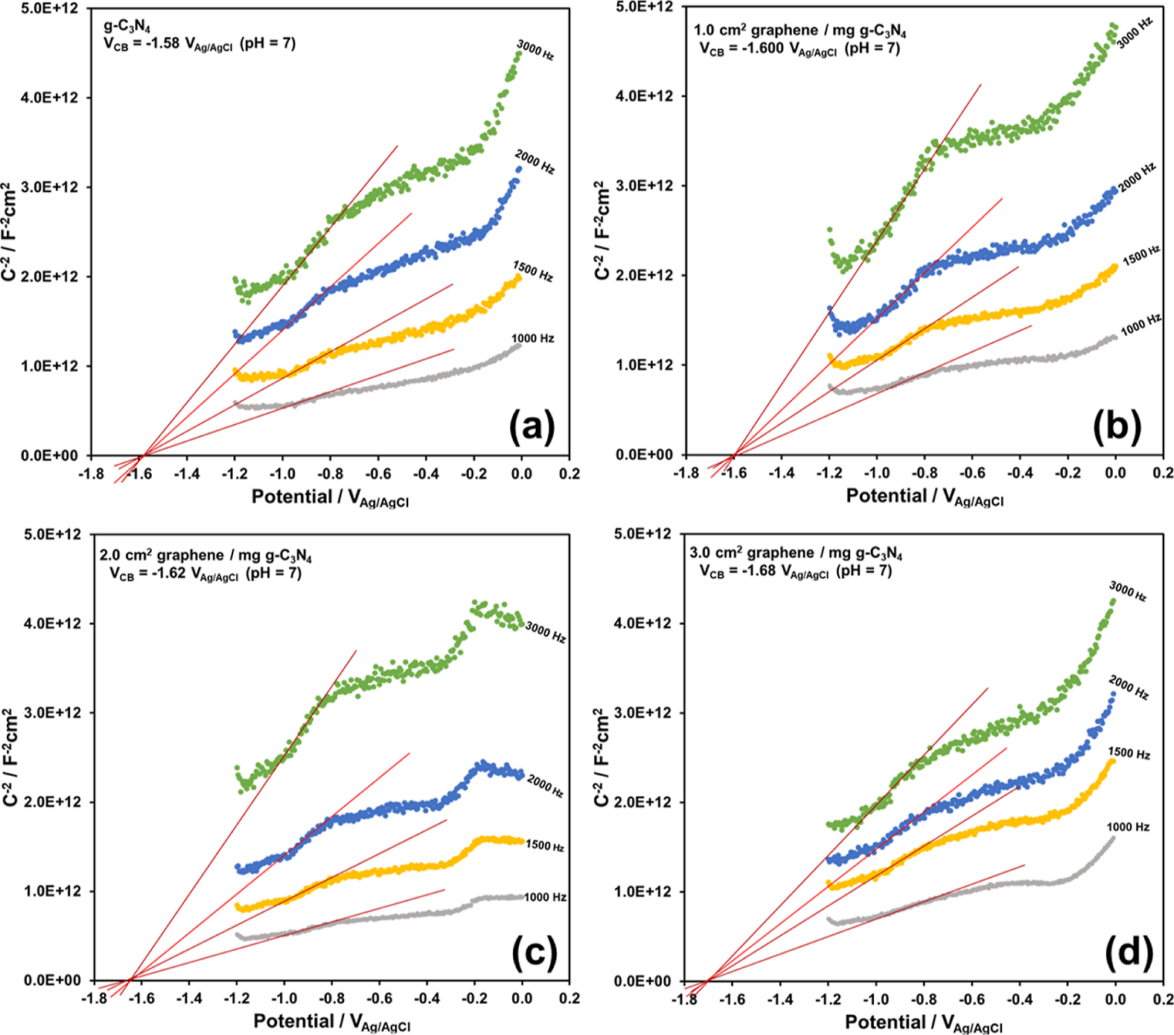
Mott–Schottky plots of (a) pure g-C_3_N_4_, (b) 1 cm^2^ graphene/mg g-C_3_N_4_, (c) 2 cm^2^ graphene/mg g-C_3_N_4_, and (d) 3 cm^2^ graphene/mg g-C_3_N_4_.

The VB edge potential of the photocatalysts directly determines the photo-oxidation power which is critical to drive I_3_^–^/I^–^ shuttle for the photo-assisted charging. If the flat band potential is assumed to be approximately equal to the CB edge potential, Mott–Schottky plots can provide the CB edge potential of the photocatalysts as in Figure 6. VB edge potential can be calculated simply by adding up the CB edge potential and optical band gap. The optical band gaps (from Figure 5), CB edge potentials (from Figure 6) and the calculated VB edge potentials are all tabulated in Table 1.

**Table 1:** *E*_g_, CB, and VB Values of Pure g-C_3_N_4_, 1 cm^2^ Graphene/mg g-C_3_N_4_, 2 cm^2^ Graphene/mg g-C_3_N_4_, and 3 cm^2^ Graphene/mg g-C_3_N_4_

	CB edge potential			
semiconductor	V_Ag/AgCl_	V_Li+/Li_	optical band gap (eV)	VB edge potential (*V*_Li+/Li_)
g-C_3_N_4_	–1.58	1.68	2.70	4.38
1 cm^2^ graphene/mg g-C_3_N_4_	–1.60	1.66	2.67	4.33
2 cm^2^ graphene/mg g-C_3_N_4_	–1.62	1.64	2.63	4.27
3 cm^2^ graphene/mg g-C_3_N_4_	–1.68	1.58	2.60	4.18

### 3.3 Photo-Assisted Charging of a Li-Ion Oxygen Battery

During the photo-assisted charging of the Li-ion oxygen battery, I^–^ ions are oxidized to I_3_^–^ ions by the photoexcited holes of the photocatalyst as in Reaction 1 as long as the VB edge potential of the photocatalyst is greater than the redox potential of the I^–^/I_3_^–^ couple (Reaction 2).^1^ All the VB edge potentials of the semiconductors tabulated in Table 1 are greater than the redox potential of Reaction 2 that they are all thermodynamically convenient photocatalysts to be used in the photo-assisted charging of the Li-ion oxygen battery.
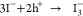
1
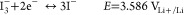
2

The generated I_3_^–^ ions diffuse to the oxygen electrode (cathode) and spontaneously oxidize low-conducting Li_2_O_2_ to Li^+^ ions and O_2_ (Reaction 3), while they are reducing back to I^–^ ions (Reaction 2) to complete the shuttle since the redox potential of Reaction 2 is greater than that of Reaction 3.
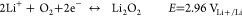
3

If the photoexcited electrons of the photocatalyst are efficiently separated from the photoexcited holes, then they flow to the anode to reduce the Li^+^ ions on the anode surface (as schematically depicted in the graphical abstract) and the overall result is that the charge potential of the Li-ion oxygen battery is compensated by the generated photovoltage.^1^ Thermodynamically, the reduction extension in the charging potential of the Li-ion oxygen battery is directly related to the level of CB edge potential that the lower the CB edge potential, the lower the charging potential.^2^ Despite the thermodynamic conveniences of all the tabulated semiconductors in Table 1, the 3 cm^2^ graphene/mg g-C_3_N_4_ nanocomposite has the highest photoanodic currents in Figure 3 since the incorporation of the graphene films into the g-C_3_N_4_ structure improves the light-harvesting capability of the g-C_3_N_4_.

**Figure 7:**
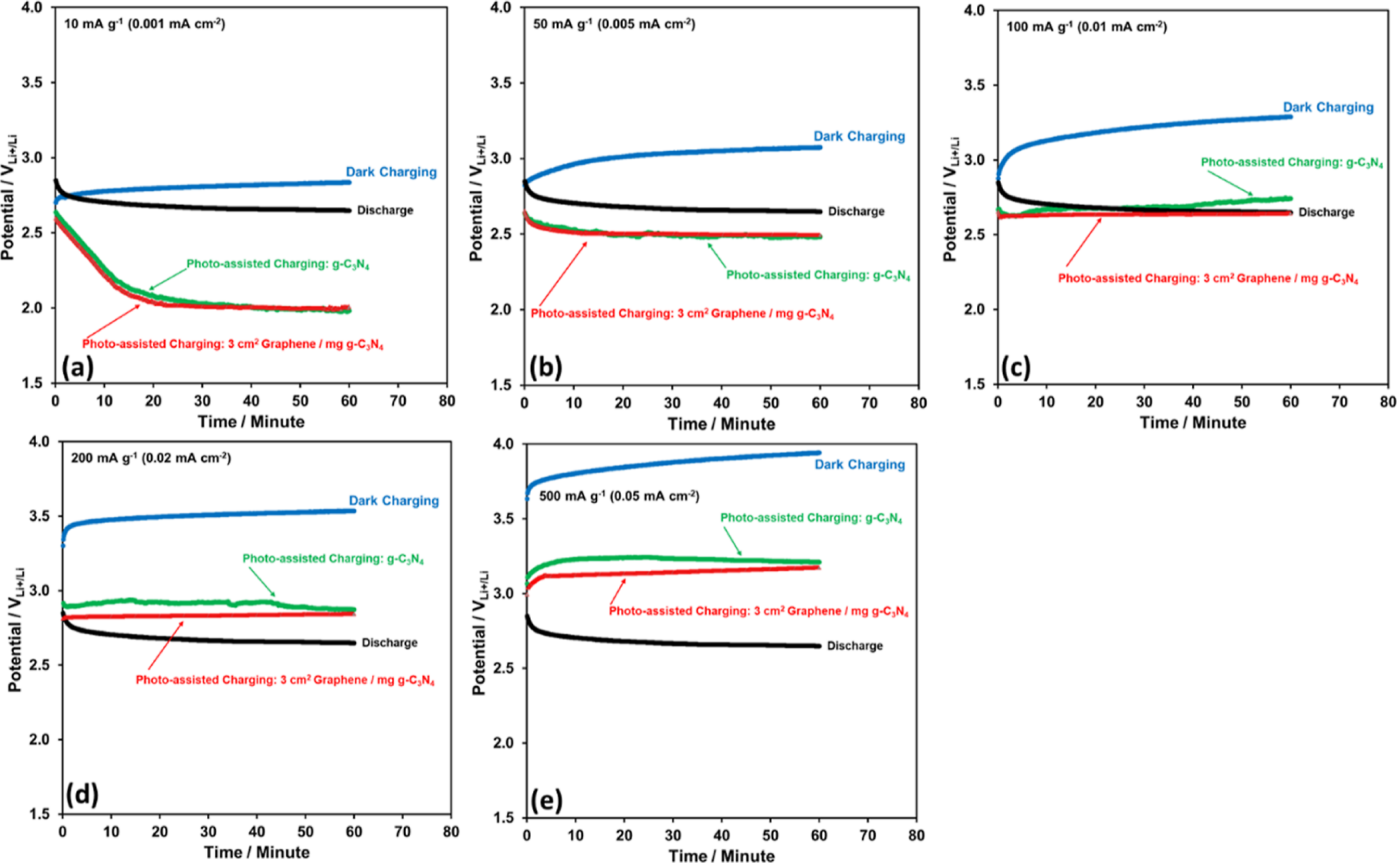
One hour long charge curves of the Li-ion oxygen battery with the g-C_3_N_4_ and 3 cm^2^ graphene/mg g-C_3_N_4_ photocatalysts under the photo-assisted charging conditions at (a) 10 mA g^–1^ (0.001 mA cm^–2^), (b) 50 mA g^–1^ (0.005 mA cm^–2^), (c) 100 mA g^–1^ (0.01 mA cm^–2^), (d) 200 mA g^–1^ (0.02 mA cm^–2^), and (e) 500 mA g^–1^ (0.05 mA cm^–2^) current densities. The discharge and dark-charging curves are also provided for comparison.

The g-C_3_N_4_ and 3 cm^2^ graphene/mg g-C_3_N_4_ nanocomposite photoelectrodes are prepared and 1 h-long charge curves at various current densities are obtained as in Figure 7 in order to observe the contribution of the graphene to the photo-assisted charging performance of the Li-ion oxygen battery. The discharge and dark-charging curves are also provided in Figure 7 to show clearly the effectiveness of photo-assisted charging. The photo-assisted charge potentials in the presence of both photoelectrodes decrease down to 2 V_Li+/Li_ at 10 mA g^–1^ (0.001 mA cm^–2^) in Figure 7a and they remain still below the discharge potential (2.65 V_Li+/Li_) at 50 mA g^–1^ (0.005 mA cm^–2^) in Figure 7b. Upon increase in the current density to 100 mA g^–1^ (0.01 mA cm^–2^) in Figure 7c, the photo-assisted charge curve in the presence of g-C_3_N_4_ photoelectrode passes above the discharge curve while that in the presence of 3 cm^2^ graphene/mg g-C_3_N_4_ photoelectrode still in the scale range of the discharge curve. Further increases in the current densities to 200 mA g^–1^ (0.02 mA cm^–2^) and 500 mA g^–1^ (0.05 mA cm^–2^), in Figure 7d,e, respectively, cause a clear separation of the photo-assisted charge curves. Obviously, the incorporated graphene films show their contribution better especially at higher current densities probably due to the increase in both conductivity and visible light absorbance efficiency of the g-C_3_N_4_ (Figures 4 and 5).

**Figure 8:**
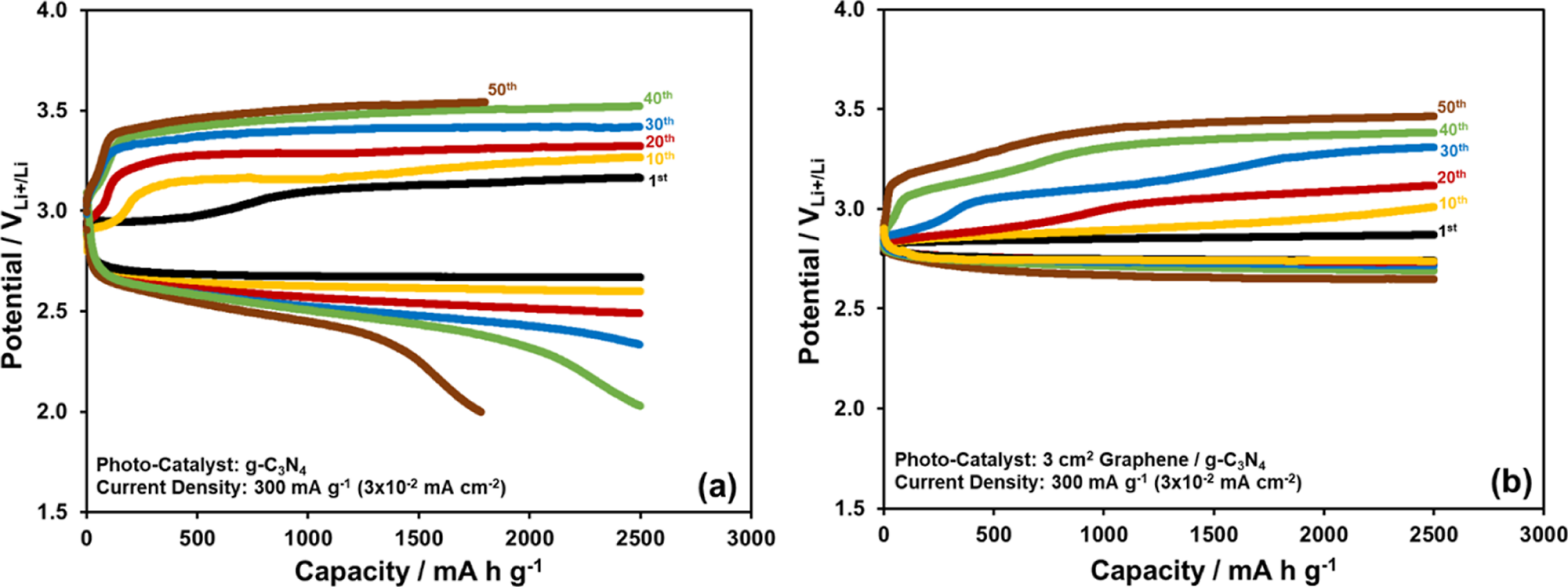
Discharge and charge curves gathered at 2500 mA h g^–1^ (0.25 mA h cm^–2^) constant capacity and 300 mA g^–1^ (3 × 10^–2^ mA cm^–2^) current density for the Li-ion oxygen battery with the (a) g-C_3_N_4_ and (b) 3 cm^2^ graphene/mg g-C_3_N_4_ photocatalysts under the photo-assisted conditions.

The photocatalytic efficiencies of both g-C_3_N_4_ and 3 cm^2^ graphene/mg g-C_3_N_4_ nanocomposite are compared by the cyclic charge/discharge test of the Li-ion oxygen battery at a constant capacity of 2500 mA h g^–1^ (0.25 mA h cm^–2^) for 50 cycles under the photoassistance as in Figure 8 (for the clear presentation, only 1st, 10th, 20th, 30th, 40th, and 50th curves are provided). The redox potential of the I^–^/I_3_^–^ couple (3.586 V_Li+/Li_) acts as a cutoff potential, which is slightly less than the theoretical potential in the used electrolyte, for the charging, and when the charge curve reaches this potential, the charging stops. In the presence of pure g-C_3_N_4_ as a photocatalyst, the charge curve reaches the cutoff potential at around the 40th cycle, and as the cycle number increases further, the discharge capacity decreases below 2500 mA h g^–1^ (0.25 mA h cm^–2^). It is reduced to 1780 mA h g^–1^ (0.178 mA h cm^–2^) at the 50th cycle. In the presence of the 3 cm^2^ graphene/mg g-C_3_N_4_ nanocomposite as a photocatalyst, however, the charge curves are still below the cutoff potential and thus there is no reduction in the discharge capacity at the 50th cycle in Figure 8. Obviously, the positive contributions (such as an increase in the conductivity and the light absorbance efficiency) of the incorporation of the graphene into the g-C_3_N_4_ structure cause the Li-ion oxygen battery to have improved performance under the illuminated conditions.

Effective photo-assisted charging requires a photocatalyst with improved properties. Thermodynamically, the VB edge potential of the photocatalyst should be greater than the redox potential of the I^–^/I_3_^–^ couple (3.586 V_Li+/Li_) to drive redox reaction shuttling, and the CB edge potential of the photocatalyst should be as low as possible to suppress the charge potential. This work shows that in addition to the thermodynamic constraints, increased conductivity, surface area, and light absorption capability are required for the photocatalyst to effectively reduce the charge potential under photoassistance, especially at the high current densities.

## 4 Conclusions

In conclusion, the g-C_3_N_4_/graphene, which was initially synthesized by CVD, nanocomposites were synthesized by thermal reduction as the photocatalysts for the photo-assisted charging of the Li-ion oxygen battery. Optical characterizations showed that the presence of graphene reduced the optical band gaps of the nanocomposites and improved the visible light utilization of g-C_3_N_4_. The positive contribution of the graphene films to the photocatalytic efficiency of g-C_3_N_4_ was also observed in the photo-assisted charging tests of the Li-ion oxygen battery that the charging potential was reduced considerably even at the high charging current densities.

## Supporting Information Available

CVD synthesis procedure of the graphene films; Raman spectra and SEM, TEM, and AFM images of the graphene films; and SEM images of the graphene/g-C_3_N_4_ nanocomposites (PDF)
