# Supplementary material for: Photocatalytic Efficiency of g-C3N4/Graphene Nanocomposites in the Photo-Assisted Charging of the Li-Ion Oxygen Battery
Source: ACS Omega. 2023 Nov 23;8(48):46227–35. doi: 10.1021/acsomega.3c07546 (PMC10702475; doi:10.1021/acsomega.3c07546)
Supplement: Supplementary file 1 — ao3c07546_si_001.pdf [file ao3c07546_si_001.pdf]

Supporting Information

Photo-Catalytic Efficiency of g-C_3N_4 / Graphene Nanocomposite in the Photo-Assisted Charging of Li-Ion Oxygen Battery

Nilay Kaçar[¥], Meltem Çayirli, Reşat Can Özden, Ersu Lökçü and Mustafa Anik*

Department of Metallurgical and Materials Engineering, Eskisehir Osmangazi University, 26040, Eskisehir, Turkey

[¥] Current Address: Department of Metallurgical and Materials Engineering, Bursa Technical University, 16310, Bursa, Turkey

*Corresponding author : manik@ogu.edu.tr

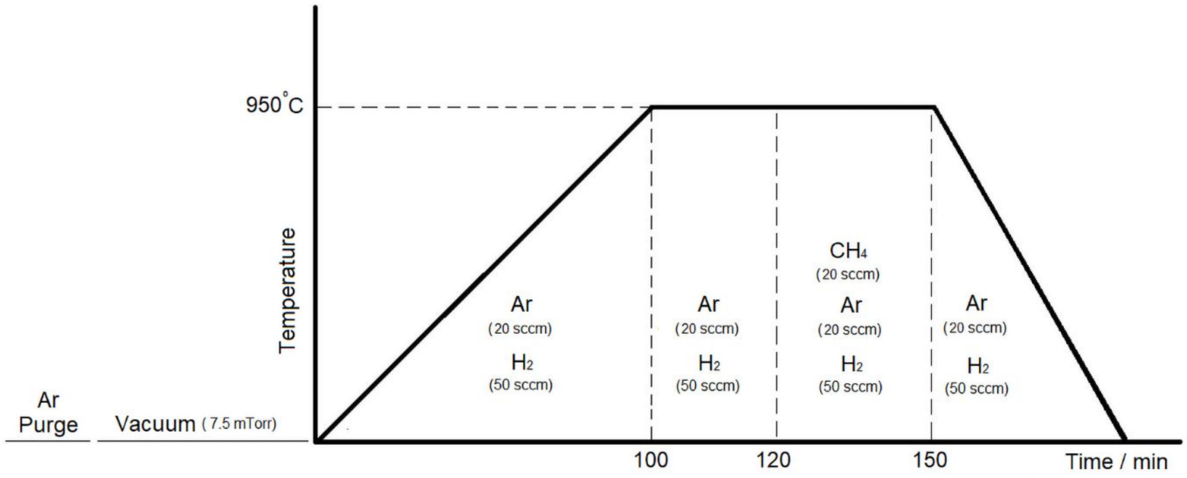

Figure S1. The synthesis procedure of the graphene films by CVD.

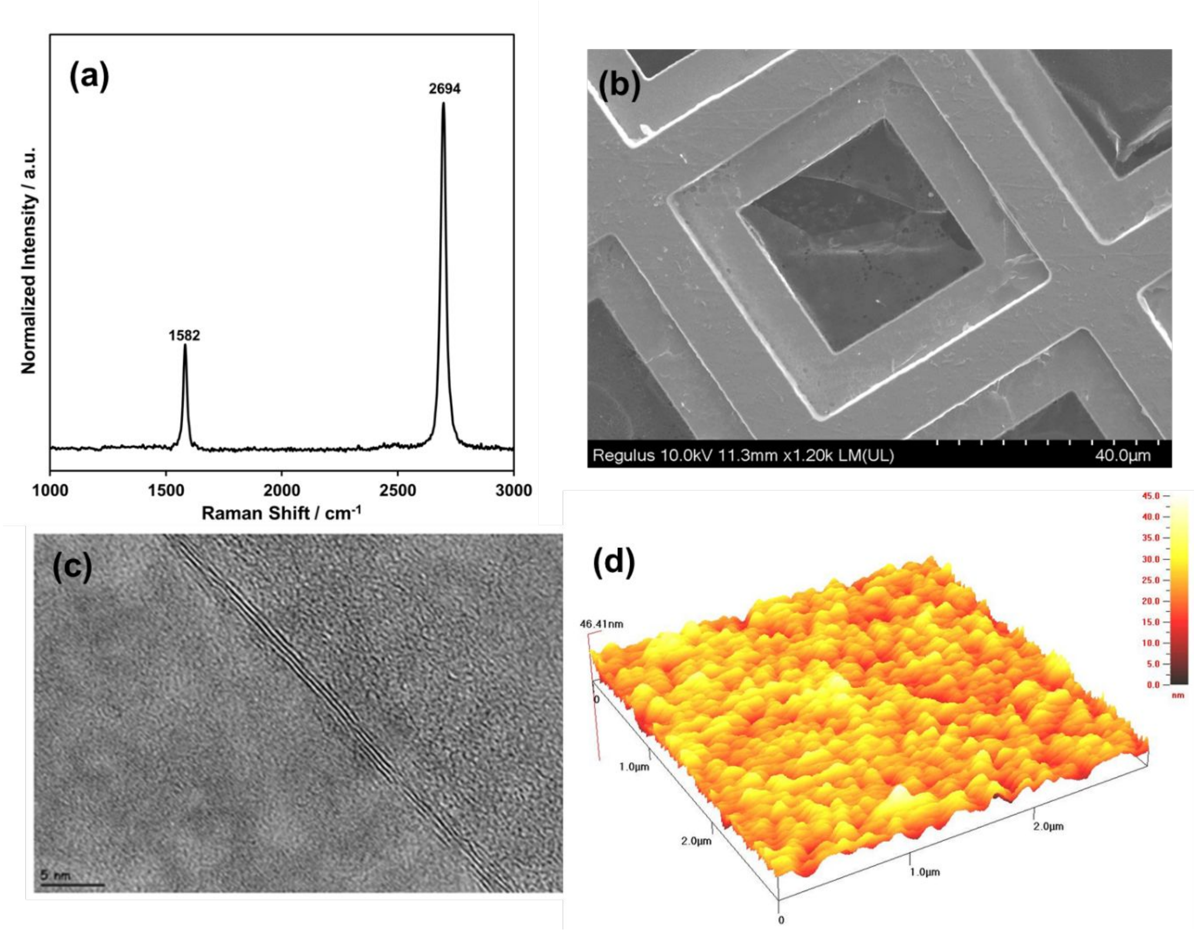

Figure S2. The (a) Raman spectra, (b) SEM, (c) TEM and (d) AFM images of the synthesized graphene films.

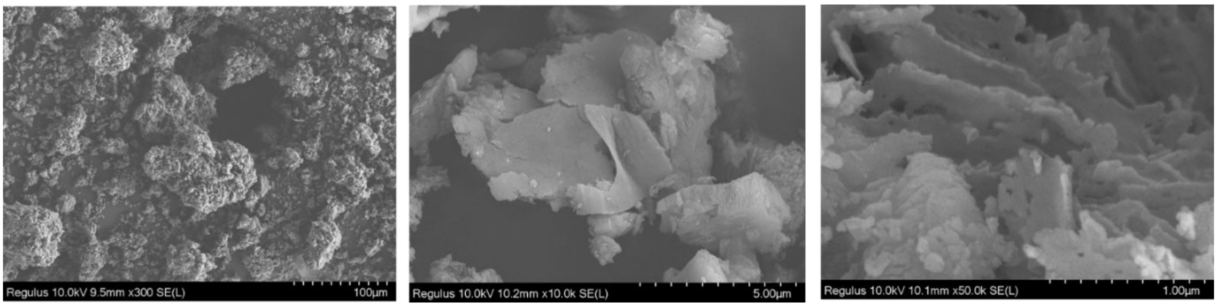

Figure S3. The morphologies of the synthesized graphene / g-C_3N_4 nanocomposites.
